# The impact of peer-assisted learning combined with scenario simulation on the trauma first aid skills and comprehensive abilities of emergency department interns

**DOI:** 10.3389/fpubh.2025.1666950

**Published:** 2025-11-25

**Authors:** Jing Fu, Yi Li

**Affiliations:** Emergency Department, Sichuan Academy of Medical Sciences & Sichuan Provincial People’s Hospital, Chengdu, Sichuan, China

**Keywords:** emergency department interns, peer-assisted learning, scenario simulation, trauma first aid skills, comprehensive abilities

## Abstract

**Background:**

This study aims to investigate the efficacy of peer-assisted learning amalgamated with scenario simulation in enhancing the trauma first aid competencies and holistic abilities of emergency department interns.

**Methods:**

Ninety interns received blended training integrating peer-assisted learning with scenario simulation (experimental group), while 90 interns from the previous year underwent traditional instructor-led training (control group). Both groups completed 10 sessions over 4 weeks, with equivalent total instructional time. Trauma first aid performance and clinical comprehensive abilities were compared between groups and within the experimental group before and after training.

**Results:**

Before training, no significant differences were observed between the two groups in any assessment items (*p* > 0.05). After training, the experimental group achieved significantly higher scores in hemostatic bandaging, fracture fixation, spinal injury management, and team cooperation (all *p* < 0.05), while differences in cardiopulmonary resuscitation and defibrillation remained non-significant. Within the experimental group, all procedure scores improved markedly after training (*p* < 0.05). Clinical comprehensive ability scores, including critical, systemic, and evidence-based thinking, were also higher than those of the control group (*p* < 0.05).

**Conclusion:**

Integrating peer-assisted learning with scenario simulation enhances trauma first aid performance and clinical reasoning among emergency department interns. This blended approach promotes teamwork, communication, and decision-making, providing a practical and effective framework for emergency medical education.

## Introduction

Trauma patients present with complex and rapidly evolving conditions, often leading to high mortality and disability rates (1–3). The timeliness of trauma care directly influences the treatment success rate and is intrinsically tied to patient safety (4). Accurate recognition of severe trauma conditions and prompt emergency management within the “golden hour” epitomizes the overall competence of emergency department physicians (5, 6). Procedures such as cardiopulmonary resuscitation (CPR) demand both technical precision and rapid clinical decision-making. However, opportunities for hands-on practice in trauma first aid skills are often limited due to ethical and logistical constraints, posing significant pedagogical challenges in balancing theoretical instruction with operational mastery. Interns, amid their professional transition phase, frequently lack adequate trauma first aid knowledge and practical experience. High-quality mentoring during this phase can significantly impact their future careers, underscoring the importance of robust intern mentoring programs. While there is a growing emphasis on trauma first aid training among emergency department staff in hospitals (7), literature on training emergency department interns in trauma first aid remains scarce.

Peer-assisted learning is emerging as an effective strategy in medical education, which emphasizes collaborative engagement, mutual feedback, and reflective learning among learners in similar conditions (8). Focusing on the principles of cognitive and social congruence, peer-assisted learning provides a safe and collaborative environment for students to learn knowledge through active explanation, observation, and feedback (9). This approach significantly improves medical students’ academic performance, particularly in the acquisition of practical clinical skills, and its effects are sustained beyond the immediate training period (10). Scenario-based learning, on the other hand, represents a learner-centered instructional approach based on situated learning theory, in which knowledge is acquired within authentic clinical contexts that mimic real-world complexity and decision-making processes (11). Scenario-based learning provides the learners with structured yet contextually rich scenarios that integrate procedural knowledge, interpersonal communication, and ethical reasoning, thereby enhancing theoretical understanding and practical application (12). This method enhances learners’ self-confidence, satisfaction, and academic performance by promoting initiatives and reflection (13). A peer-assisted and scenario-based simulation model has proven to be effective in improving students’ clinical knowledge, teamwork, communication, and understanding of patient perspectives (14). This hybrid model combines the active participation and reflective feedback of peer-assisted learning to enhance both problem-solving ability and collaborative learning outcomes. However, empirical evidence on the effectiveness of such combined methods specifically for emergency department interns remains scarce.

To address this gap, our emergency department implemented a blended educational model combining peer-assisted learning and scenario-based simulation, designed to enhance both procedural skills and teamwork dynamics. Previous reports suggest that such immersive training can bridge the theory-practice divide, yet quantitative evidence regarding its effectiveness for emergency department interns remains limited. Addressing the transition of interns to clinical roles and strengthening their trauma first aid skills and comprehensive abilities is a pressing issue in emergency department education and training. Accordingly, this study aimed to evaluate the effectiveness of a peer-assisted learning combined with scenario-based simulation model for trauma first aid education. We hypothesized that interns who received this blended training would demonstrate (1) higher proficiency in trauma first aid procedures, (2) improved teamwork and communication skills, and (3) greater overall clinical competence compared with those receiving traditional instruction.

## Materials and methods

### Research subjects

The experimental group consisted of 90 doctors interning in our emergency department from August 2022 to August 2023. These interns were final-year undergraduate medical interns completing mandatory clinical rotations, about 1–1.5 years, as part of their degree requirements, who had not yet graduated or obtained medical licensure and performed clinical tasks under direct supervision. They were divided into 10 groups and participated in peer-assisted learning combined with scenario simulation courses. Each course integrated 2–3 related operational items based on scenario cues, with 10 courses conducted in total. The control group included 90 doctors interning in the same department from July 2021 to July 2022, who were similarly final-year undergraduate interns receiving traditional single-operation training, with each course training on a single item, totaling 10 courses. This study was approved by the hospital’s ethics committee.

### Preparation of instructors

A team of senior attending physicians from the emergency and orthopedic departments with extensive clinical and teaching experience was assembled. Prior to course initiation, instructor training was conducted, encompassing methodologies of peer-assisted learning integrated with scenario simulation, and course arrangements. Instructors were finalized post collective lesson preparation and preliminary lectures. Pre-course, instructors designed scenario cases based on teaching content, each case entailing 1–2 condition variations tailored to learners’ operations. Assistant instructors set up the scenario environment and prepared necessary materials accordingly. Learners were required to preview theoretical skills relevant to the course, and upon successful assessment, were enrolled in the course. Learners were grouped into operational teams of nine, totaling 10 groups. Instructors facilitated scenario progression based on designed cases, integrating operational tasks therein. Learners analyzed and made preliminary diagnoses before executing related operations. Post-operation, instructors collected feedback, guiding learners to refine operational procedures. Each teaching session spanned four academic hours.

### Teaching methods

Interns in the experimental group received scenario simulation-based training. To ensure consistency across groups, all scenario simulations were developed in advance using standardized case templates that included patient background, clinical progression, and key decision points. Prior to the study, the teaching team underwent an internal meeting to standardize course materials, ensuring that all trainees received comparable learning experiences. Using cardiopulmonary resuscitation as an illustrative example, the teaching protocol proceeded as follows: After theoretical instruction, interns participated in simulated CPR scenarios within a clinical skills training center. The classroom was arranged to replicate a surgical consultation room equipped with a CPR training manikin, while an adjacent area simulated a preparation room containing an emergency cart and defibrillator. Interns were divided into 10 groups, with nine interns per group, each assigned specific roles including a physician providing treatment, a nurse, a patient’s family member, and additional physicians or nurses within the simulated environment. The instructor presented a clinical scenario involving an elderly patient with underlying cardiovascular disease who suddenly lost consciousness during a bedside consultation. Interns were tasked with organizing an emergency response. Each group first conducted independent discussions, followed by an instructor-led review of standardized CPR protocols, operational steps, and live demonstrations. During simulations, the designated physician assessed the manikin’s vital signs to confirm cardiac arrest, activated the in-hospital emergency response system, and coordinated team efforts to retrieve critical equipment. One physician assumed the role of team leader, assessing the manikin’s vital signs, activating the emergency response system, and coordinating resuscitation efforts. Two physicians performed chest compressions and rescue breathing, while a nurse managed defibrillator preparation and intravenous access. Others nurse communicated with the patient’s family member to provide updates and address concerns. Following each simulation, interns rotated roles to experience diverse clinical responsibilities and reinforce teamwork dynamics. Instructors provided real-time feedback to correct procedural inaccuracies, ensuring all participants mastered the CPR workflow.

Interns in the control group received traditional didactic training. Instructors delivered theoretical lectures, demonstrated technical procedures such as CPR, defibrillation, hemostatic bandaging, fracture immobilization, and spinal injury management, and supervised interns during practice sessions without incorporating role-playing or scenario simulations.

The training of experimental and control groups is both consisted of 10 sessions delivering over a four-week period, with two to three sessions conducted each week. Each session lasted approximately 2 h. All interns completed the full series of 10 sessions under the supervision of the same teaching team to ensure consistency of instruction and assessment.

### Classroom assessment

The assessment was conducted through non-complex scenario simulations designed solely as procedural context, distinct from educational simulations that incorporate multifaceted backgrounds or emergent events. The evaluation of trauma first aid skills was conducted in accordance with the *National Standard for the Clinical Practice Competency Assessment of Resident Physicians (2022 edition)*, issued by the Health Human Resources Development Center, National Health Commission of the People’s Republic of China. This standardized framework specifies operational criteria and performance levels for key emergency procedures, including cardiopulmonary resuscitation, defibrillation, hemostatic bandaging, fracture fixation, and spinal injury management.

Each learner group randomly selected standardized task prompts such as cardiopulmonary resuscitation, defibrillation, hemostatic bandaging, fracture external fixation, and spinal injury management. The assessment was conducted on-site by three evaluators, and the final score was calculated as the mean of their individual assessments using a blind method. Task performance was scored based on adherence to standard operational steps, while team cooperation was assessed through clear role division, timely error correction, effective communication, and composed decision-making. Scoring criteria were delineated as follows: Poor (0 points) for failure to execute or incorrect execution of the task; Fair (5 points) for performance between poor and average; Average (10 points) for partial execution or partial incorrect execution of the task; Good (15 points) for performance between average and very good; Very Good (20 points) for correct execution of the task.

### Assessment of clinical comprehensive ability

Prior to and following the training, the clinical thinking ability of emergency department interns was assessed using a scale adapted from the Nursing Critical Thinking in Clinical Practice Questionnaire (N-CT-4 Practice) and improved in later studies (15–17). The adapted version in this study included three domains, including systemic thinking, evidence-based thinking, and critical thinking, with a total of 24 items rated on a 5-point Likert scale (1–5 points). Higher scores indicated stronger clinical reasoning and comprehensive ability.

### Statistical methods

Data were analyzed using the SPSS 25.0 software package. Quantitative data conforming to a normal distribution were presented as $\left(\bar{Χ}\pm \;S\right)$, and intergroup comparisons were conducted through *t*-test analysis. Categorical data were represented by rates (%), with intergroup comparisons performed through *χ^2^* test. A *p*-value of <0.05 was considered statistically significant.

## Results

### General information comparison of emergency department interns across both groups

The comparison of general information among emergency department interns across both groups revealed no statistically significant differences (*p* > 0.05), as depicted in Table 1. Regarding the education categories in Table 1, in the Chinese medical education system, ‘three-year college’ refers to vocational medical programs in junior college that focus on practical clinical skills and train mid-level healthcare professionals, while ‘undergraduate course’ represents a comprehensive five-year medical program leading to a Bachelor of Medicine degree.

**Table 1:** Comparison of general information of emergency department interns across both groups.

Group	Number of participants	Gender		Age (years)	Education	
		Male	Female		Three-year college	Undergraduate course
Experimental group	90	48	42	22.6 ± 2.0	10	80
Control group	90	50	40	22.0 ± 2.0	12	78
*χ*^2^/*t-*value		2.000		1.843	2.773	
*p-*value		0.157		0.067	0.096	

### Pre-course preparation scores comparison of emergency department interns across both groups

The comparison of pre-course preparation scores, encompassing cardiopulmonary resuscitation, defibrillation, hemostatic bandaging, fracture external fixation, and spinal injury handling, between the experimental and control groups revealed no statistically significant differences (*p* > 0.05), as illustrated in Table 2.

**Table 2:** Pre-course preparation scores comparison of emergency department interns across both groups $\left(\bar{Χ}\pm S\right)$.

Group	Number of participants	Cardiopulmonary resuscitation	Defibrillation	Hemostatic bandaging	Fracture external fixation	Spinal injury handling
Experimental group	90	72.3 ± 6.0	68.2 ± 8.4	71.3 ± 5.9	75.4 ± 5.3	69.0 ± 5.7
Control group	90	71.8 ± 7.0	68.4 ± 7.9	70.5 ± 7.4	75.5 ± 5.0	69.4 ± 7.3
*t-*value		−0.548	−0.119	−0.826	−0.130	0.412
*p-*value		0.585	0.906	0.410	0.897	0.681

### Comparison of post-training scores between the two groups of emergency department interns

**Figure 1:**
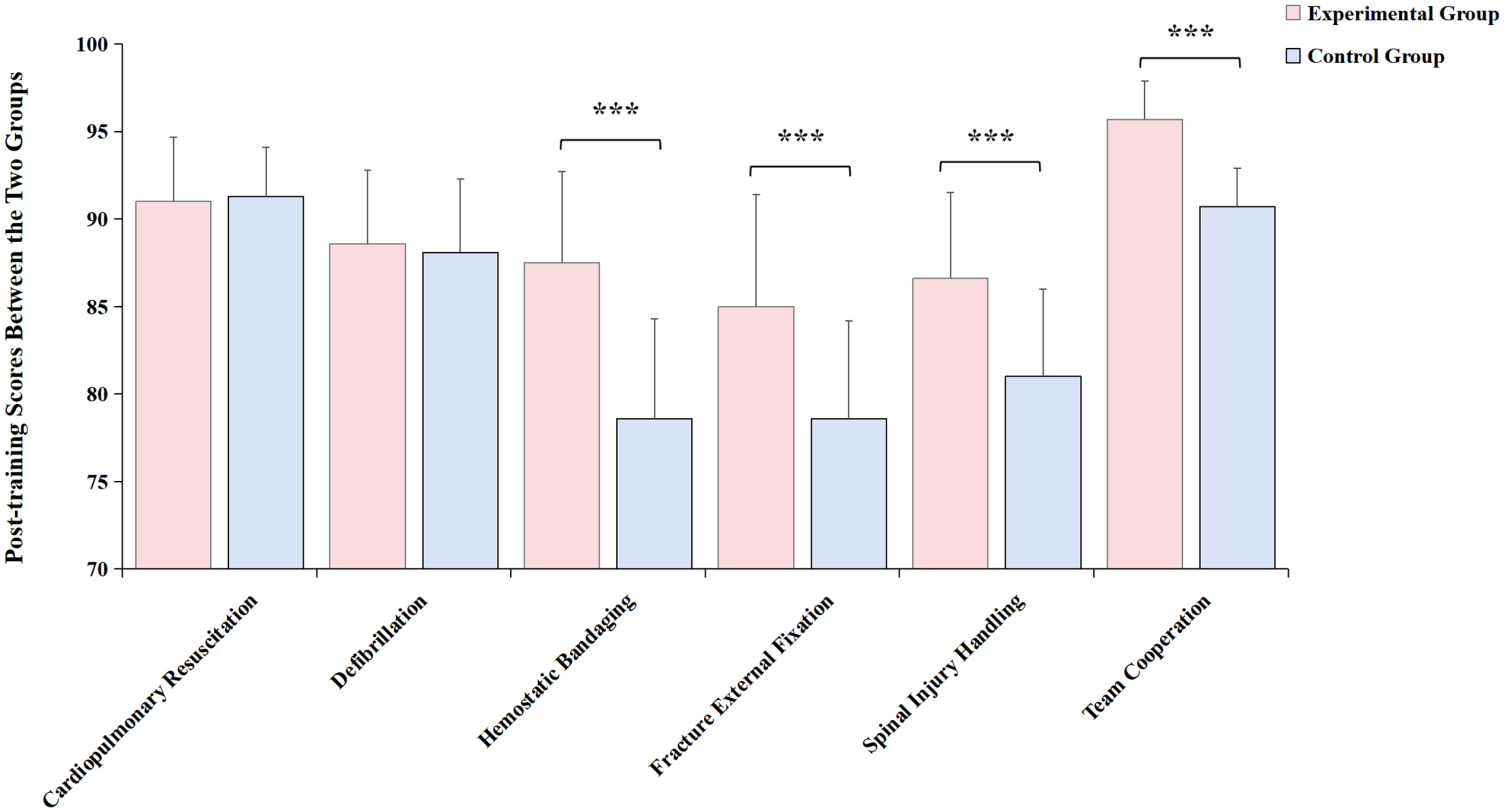
Comparison of post-training scores between the two groups of emergency department interns. Experimental group had significantly higher scores in hemostatic bandaging, fracture external fixation, spinal injury handling, and team cooperation compared to the control group. Red bar: experimental group; blue bar: control group. ^***^p < 0.001.

There were no statistically significant differences in the scores for cardiopulmonary resuscitation and defibrillation between the groups (Figure 1, both *p* > 0.05). However, the experimental group achieved higher scores in hemostatic bandaging, fracture external fixation, spinal injury handling, and team cooperation compared to the control group, with the differences being statistically significant (Figure 1, all *p* < 0.001), as detailed in Table 3.

**Table 3:** Comparison of post-training scores between the two groups of emergency department interns $\left(\bar{Χ}\pm S\right)$.

Group	Cardiopulmonary resuscitation	Defibrillation	Hemostatic bandaging	Fracture external fixation	Spinal injury handling	Team cooperation
Experimental group	91.0 ± 3.7	88.6 ± 4.2	87.5 ± 5.2	85.0 ± 6.4	86.6 ± 4.9	95.7 ± 2.2
Control group	91.3 ± 2.8	88.1 ± 4.2	78.6 ± 5.7	78.6 ± 5.6	81.0 ± 5.0	90.7 ± 2.2
*t-*value	−0.571	−0.838	11.037	−7.210	7.709	−15.189
*p-*value	0.569	0.403	<0.001	<0.001	<0.001	<0.001

### Comparison of operation scores pre- and post-training in the experimental group

**Figure 2:**
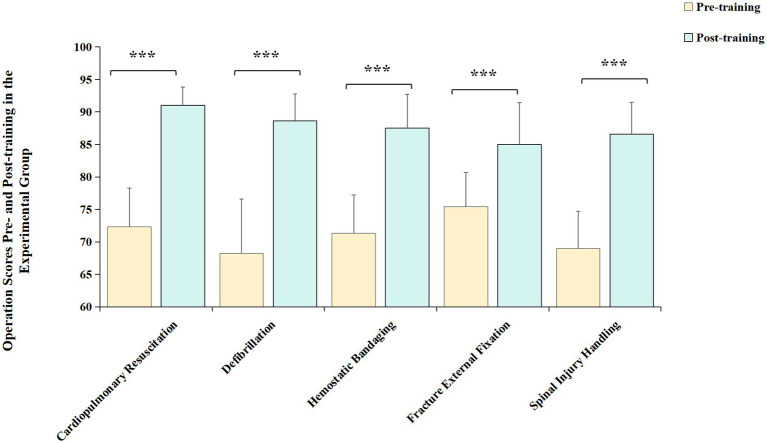
Comparison of operation scores pre- and post-training in the experimental group. The post-training scores for cardiopulmonary resuscitation, defibrillation, hemostatic bandaging, fracture external fixation, and spinal injury handling of experimental group were all significantly elevated, compared to pre-training scores. Yellow bar: pre-training scores of the experimental group; green bar: post-training scores of the experimental group. ^***^p < 0.001.

In the experimental group, post-training scores for cardiopulmonary resuscitation, defibrillation, hemostatic bandaging, fracture external fixation, and spinal injury handling were all elevated compared to pre-training scores, exhibiting statistically significant differences (Figure 2, all *p* < 0.001), as showcased in Table 4.

**Table 4:** Comparison of operation scores pre- and post-training in the experimental group $\left(\bar{Χ}\pm S\right)$.

Time	Cardiopulmonary resuscitation	Defibrillation	Hemostatic bandaging	Fracture external fixation	Spinal injury handling
Pre-training	72.3 ± 6.0	68.2 ± 8.4	71.3 ± 5.9	75.4 ± 5.3	69.0 ± 5.7
Post-training	91.0 ± 2.8	88.6 ± 4.2	87.5 ± 5.2	85.0 ± 6.4	86.6 ± 4.9
*t-*value	−25.223	−20.620	−19.660	−10.999	−22.429
*p-*value	<0.001	<0.001	0.003	<0.001	<0.001

### Comparison of post-training comprehensive abilities between two groups of emergency department interns

**Figure 3:**
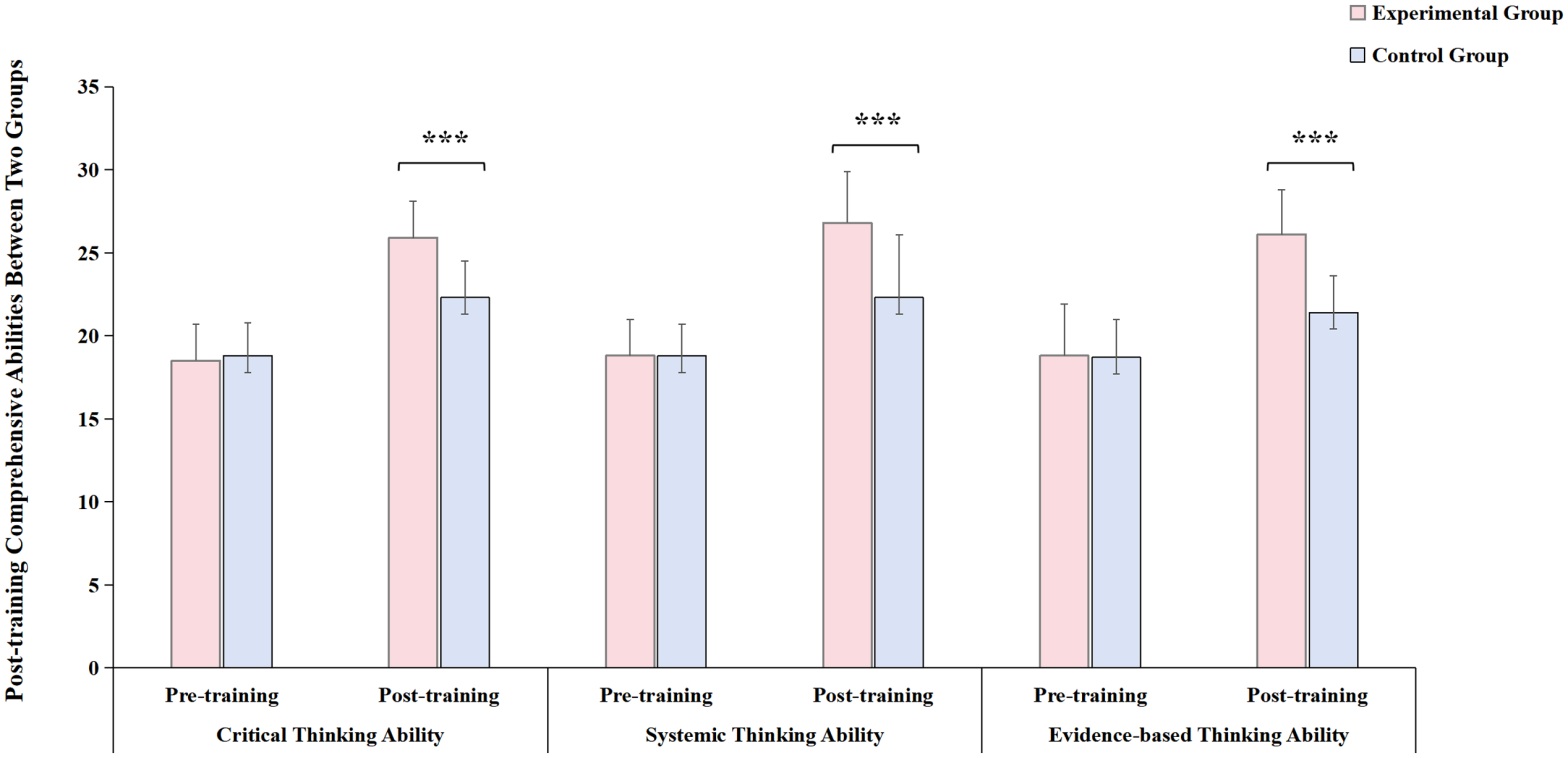
Comparison of post-training comprehensive abilities between two groups of emergency department interns. post-training, both groups demonstrated improved scores in critical thinking, systemic thinking, and evidence-based thinking abilities compared to pre-training scores, with the experimental group surpassing the control group. Red bar: experimental group; blue bar: control group. ^***^p < 0.001.

Before training, there was no statistically significant difference (*p* > 0.05) in clinical comprehensive abilities across all dimensions between the two groups of interns. Post-training, both groups demonstrated improved scores in critical thinking, systemic thinking, and evidence-based thinking abilities compared to pre-training scores, with the experimental group surpassing the control group. These differences were statistically significant (Figure 3, all *p* < 0.001), as illustrated in Table 5.

**Table 5:** Comparison of post-training comprehensive abilities between two groups of emergency department interns $\left(\bar{Χ}\pm S\right)$.

Group	Critical thinking ability		Systemic thinking ability		Evidence-based thinking ability	
	Pre-training	Post-training	Pre-training	Post-training	Pre-training	Post-training
Experimental group	18.5 ± 2.2	25.9 ± 2.2	18.8 ± 2.2	26.8 ± 3.1	18.8 ± 3.1	26.1 ± 2.7
Control group	18.8 ± 2.0	22.3 ± 2.2	18.8 ± 1.9	22.3 ± 3.8	18.7 ± 2.3	21.4 ± 2.2
*t-*value	−1.091	11.021	0.036	8.658	−0.434	12.835
*p-*value	0.277	<0.001	0.971	<0.001	0.665	<0.001

## Discussion

Emergency trauma patients exhibit characteristics of suddenness, urgency, and complexity, demanding clinicians to possess high levels of cognitive agility, technical proficiency, and rapid-response capability (18, 19). Traditional trauma simulation training in domestic settings primarily adopts a “demonstration-practice-assessment” three-step approach, often focusing on single-skill operations with less emphasis on team coordination based on case scenarios. Notably, conventional medical pedagogy has long relied on didactic “cramming” or “teacher-centered” methods, where instructors unilaterally deliver theoretical knowledge, relegating students to passive recipients. This approach stifles learning motivation, suppresses critical thinking, and confines cognitive development to basic levels of memorization and application, while limiting opportunities for students to articulate ideas or engage in higher-order analysis.

However, clinical scenarios are often complex. Frontline resident physicians are frequently required to first assess the patient’s condition, make a preliminary diagnosis, and then decide on a course of action (19, 20), including task allocation, seeking assistance, and clinical operations, which are abilities not fortified through single-skill operation training. This leads to a lack of effective engagement in interns’ learning enthusiasm, rendering training outcomes suboptimal, and necessitating the exploration of more proactive and effective training methods (21–23). While both peer-assisted learning and scenario-based simulation have demonstrated strong educational value in this case, each method also presents inherent limitations when applied independently. Scenario-based simulation emphasizes experiential learning in realistic contexts but can be overly teacher-centered that the learners may take the actions correctly during simulating courses following guidance, but fail to put them into real-world practice during emergency events (24). On the other hand, peer-assisted learning promotes active participation and feedback, provides emotional support and reduces anxiety, improves critical thinking and problem-solving skills, and a psychologically safe environment for reflection between the learners (25). However, its effectiveness might be related to the differences in peers’ teaching experience and clinical proficiency (26). Therefore, peer-assisted learning combined with scenario simulation training is carried out through role demonstrations, utilizing multimedia and simulation equipment to construct a realistic medical environment. The scenario-based component provides realistic, high-stakes contexts for practicing trauma first aid, while the peer-assisted structure encourages active reflection, communication, and collaborative problem-solving. In recent years, with the changes in medical practice environments and the development of medical simulation education, medical scenario simulation education has gradually been promoted domestically, fostering and enhancing multifaceted work capabilities and levels of interns (14). The results of this study demonstrate that this case-based peer-assisted learning combined with scenario simulation course has achieved favorable educational outcomes.

The comparison of pre-training preparatory scores between the experimental group and the control group showed no statistical significance. Post-training score comparison revealed that in terms of “Team Cooperation,” the scores of the experimental group were superior to those of the control group. In the “Project Process and Operations” aspect, among the five assessment items, the scores for CPR and Defibrillation showed no statistical difference when compared to traditional methods. This may be attributed to the fact that CPR is the most common basic emergency skill and a mandatory and assessable content at various learning stages. The participants have a good foundation in it, and the requirement for team coordination is relatively low, hence traditional skills operation training could also achieve good training effects (3). For Hemorrhage Control, Fracture Immobilization, and Spinal Injury Transport, three items, the scores of the experimental group were higher than those of the control group. These three items require better coordination among team members (2), especially Spinal Injury Transport, indicating that the teaching method of the experimental group has better training effects on skills operation that requires multi-person coordination and emphasizes team cooperation. Moreover, the fact that the scores of the experimental group after training were significantly higher than before training also affirmed the effectiveness of the Trauma Peer-Assisted Learning Combined with Scenario Simulation course.

Several limitations of this study should be acknowledged. First, this study was conducted in a single hospital, without incorporating multi-center data, which may be limited by the institution’s teaching resources, faculty expertise, or patient demographics. Second, although the emergency skills assessment was conducted using a blind method, it relied to some extent on the subjective judgment of instructors, which could potentially introduce scoring bias. Thirdly, while the total duration of each session was controlled at approximately 2 h for both groups, minor variations may have occurred due to the interactive discussions in the peer-assisted learning group. Although this time difference was minimal, it could still be considered a potential confounding factor influencing learning outcomes. The present findings may not be directly generalizable to other institutions, as the differences in the characteristics of our institution, such as its teaching facilities, number of instructors, and local training culture that may not fully represent other hospitals or teaching settings. Future research should include multiple hospitals with different teaching systems to confirm whether the same training model produces consistent results. Including multiple hospitals with different teaching systems and standardized time allocations are also vital to further verify the generalizability of the training model. Additionally, including more emergency department interns could have provided more comprehensive research findings.

In summary, the mentoring approach of Peer-Assisted Learning Combined with Scenario Simulation effectively enhances the trauma first aid skills and clinical comprehensive capabilities of emergency department interns. This method amplifies the mentoring outcomes, aids medical teams in promptly implementing accurate rescue measures, and elevates the success rate of rescues. It can be adopted as a routine long-term course for mentoring emergency department interns.

## Data Availability

The original contributions presented in the study are included in the article/supplementary material, further inquiries can be directed to the corresponding author.
